# Faster title and abstract screening? Evaluating Abstrackr, a semi-automated online screening program for systematic reviewers

**DOI:** 10.1186/s13643-015-0067-6

**Published:** 2015-06-15

**Authors:** John Rathbone, Tammy Hoffmann, Paul Glasziou

**Affiliations:** Centre for Research in Evidence-Based Practice, Bond University, Gold Coast, Australia

## Abstract

**Background:**

Citation screening is time consuming and inefficient. We sought to evaluate the performance of Abstrackr, a semi-automated online tool for predictive title and abstract screening.

**Methods:**

Four systematic reviews (aHUS, dietary fibre, ECHO, rituximab) were used to evaluate Abstrackr. Citations from electronic searches of biomedical databases were imported into Abstrackr, and titles and abstracts were screened and included or excluded according to the entry criteria. This process was continued until Abstrackr predicted and classified the remaining unscreened citations as relevant or irrelevant. These classification predictions were checked for accuracy against the original review decisions. Sensitivity analyses were performed to assess the effects of including case reports in the aHUS dataset whilst screening and the effects of using larger imbalanced datasets with the ECHO dataset. The performance of Abstrackr was calculated according to the number of relevant studies missed, the workload saving, the false negative rate, and the precision of the algorithm to correctly predict relevant studies for inclusion, i.e. further full text inspection.

**Results:**

Of the unscreened citations, Abstrackr’s prediction algorithm correctly identified all relevant citations for the rituximab and dietary fibre reviews. However, one relevant citation in both the aHUS and ECHO reviews was incorrectly predicted as not relevant. The workload saving achieved with Abstrackr varied depending on the complexity and size of the reviews (9 % rituximab, 40 % dietary fibre, 67 % aHUS, and 57 % ECHO). The proportion of citations predicted as relevant, and therefore, warranting further full text inspection (i.e. the precision of the prediction) ranged from 16 % (aHUS) to 45 % (rituximab) and was affected by the complexity of the reviews. The false negative rate ranged from 2.4 to 21.7 %. Sensitivity analysis performed on the aHUS dataset increased the precision from 16 to 25 % and increased the workload saving by 10 % but increased the number of relevant studies missed. Sensitivity analysis performed with the larger ECHO dataset increased the workload saving (80 %) but reduced the precision (6.8 %) and increased the number of missed citations.

**Conclusions:**

Semi-automated title and abstract screening with Abstrackr has the potential to save time and reduce research waste.

## Background

Systematic reviews require a comprehensive search and appraisal of the literature to identify all relevant studies for inclusion. Typically, this involves a team of reviewers inspecting thousands of records that are produced from database searches. The large number of citations retrieved is partly due to the inadequate coding of studies indexed in biomedical databases such as MEDLINE and EMBASE. This produces imprecise search results; sometimes less than 1 % of studies screened are included in a systematic review [1, 2]. Systematic reviews have also become more time consuming due to the growth in the volume and scatter of randomised trials [3], additional reporting steps [4–6], and the incorporation of more complex methodologies such as network meta-analysis and the acquisition of clinical study reports [7]. Consequently, many systematic reviews are out of date [8, 9]. With all these challenges, there is a need to adopt techniques from computer science that can semi-automate screening in order to expedite the process of study selection.

Text mining techniques are used to identify relevant information from text using statistical pattern learning that recognises patterns in data. Typically, such patterns are learnt from labelled training data that are then applied to datasets. A common application of such techniques is used to separate spam from real emails. Pattern recognition algorithms aim to provide the most likely matching of the inputs, taking into account their statistical variation. They have been applied in a variety of ways in evidence-based medicine to expedite tasks that would otherwise be omitted due to the time and cost involved if they were performed manually. For example, text mining has been used to assess the frequency of adverse effects of drugs by analysing patient medical records [10] and to expedite scoping searches [11]. Text mining has the potential to reduce the workload of systematic reviewers by assisting with the identification of relevant trials during the title and abstract screening stage of a systematic review.

Abstrackr [12] is a free, open-source [13], citation screening program, currently at beta testing stage that uses an algorithm within an active learning framework to predict the likelihood of citations being relevant. It uses text unigrams and bigrams within the annotated abstracts for the predictive modelling. Abstrackr biases the citations so that the most relevant are prioritised for screening first. Only limited research to date has been conducted into the strengths and limitations of semi-automated citation screening [14, 15]. The aim of this study was to evaluate the performance of the Abstrackr algorithm. It was chosen for evaluation in preference to other text mining tools because existing literature indicates that the recall accuracy of Abstrackr is very high [14–17], and therefore, a promising predictive text mining tool for systematic reviews, where the primary goal is to identify all relevant studies.

## Methods

The trial citations of four systematic reviews [1, 18–20] retrieved electronically from biomedical database searches (MEDLINE, EMBASE, CINAHL, Cochrane CENTRAL) were used to evaluate Abstrackr. Three systematic reviews evaluated treatment effectiveness: dietary fibre interventions for colorectal cancer, rituximab and adjunctive chemotherapy interventions for non-Hodgkin’s lymphoma, eculizumab for atypical hemolytic uremic syndrome (aHUS), and one diagnostic accuracy review of echocardiography (ECHO) was included. Each systematic review was chosen because of their different characteristics: for example, the aHUS review included all study designs except case reports; the interventions in the rituximab review included multiply chemotherapy interventions rather than a simple drug A versus drug B comparison; the dataset from the dietary fibre review was from a specialised register which provides a more homogeneous and smaller set of citations and therefore presents a challenge to supervised machine learning algorithms because they perform better on large datasets; and the ECHO was chosen because it is a diagnostic accuracy review.

Citations were uploaded to Abstrackr, and titles and abstracts were screened for relevance by one author with relevant studies selected for inclusion and clearly irrelevant studies excluded. Screening continued until the algorithm’s stopping criterion indicated that predictions were available for viewing. This is based upon a simple heuristic requiring a set number of citations to be screened manually. The remaining unscreened citations were inspected according to the probability estimates and hard binary prediction made by the algorithm and cross-checked against the original review decisions.

The performance of Abstrackr was assessed by calculating the precision, the false negative rate, the proportion missed, and the workload saving. The precision is the percentage of citations predicted relevant by Abstrackr that are subsequently deemed relevant by the *reviewer* for further full text inspection. The false negative rate is the percentage of citations that are relevant for further full text inspection but were predicted to be irrelevant by Abstrackr. The proportion missed is the number of studies missed by Abstrackr that were included in the published reviews, out of those studies predicted to be irrelevant. The workload saving is the proportion of citations predicted irrelevant out of the total number of citations.

A post hoc sensitivity analysis was performed on the aHUS dataset because many of the included and excluded studies were methodologically similar, and therefore, excluding near matches might impede the learning algorithm. For example, case reports were originally excluded, but case series and RCTs were relevant and included. Therefore, by rescreening the aHUS dataset and also including case reports, we sought to determine if their inclusion would improve the machine learning precision by reducing superficially conflicting decisions. A post hoc sensitivity analysis was also performed on a substantially larger ECHO dataset to determine if this would affect the workload saving.

## Results

A total for four datasets from existing systematic reviews (aHUS *n* = 1415), (dietary fibre *n* = 517), (ECHO *n* = 1735) and (Rituximab *n* = 1042) were uploaded to Abstrackr and screened for relevance until the classification algorithm made predictions.

### Atypical haemolytic uremic syndrome dataset (excluding case reports)

**Fig. 1:**
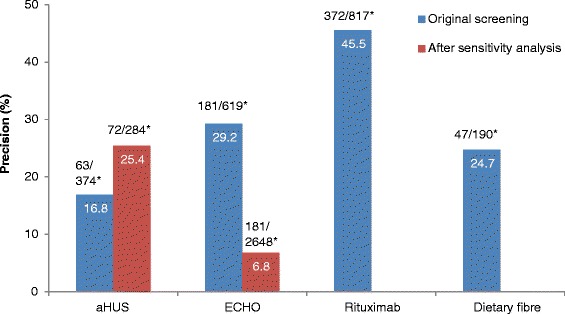
Percentage of citations predicted by Abstrackr that were relevant for further full text inspection. ^*^Raw numbers of the proportion of citations selected for inspection

**Fig. 2:**
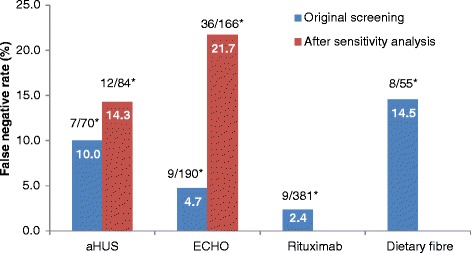
False negative rate. *Raw numbers of the proportion of citations incorrectly predicted by Abstrackr to be irrelevant for further inspection

**Fig. 3:**
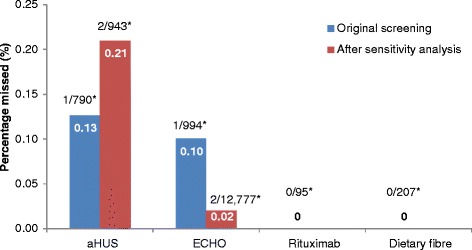
Percentage of studies missed by Abstrackr—but were included in the reviews. ^*^Raw numbers of the proportion of citations missed (predicted not relevant)

**Fig. 4:**
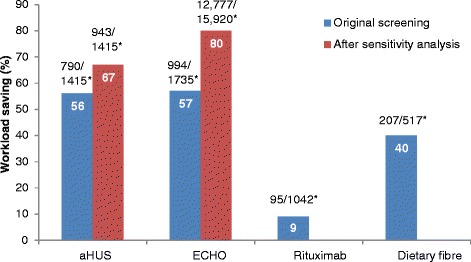
Workload saving (%) when using Abstrackr in each of the four datasets. ^*^Raw numbers of the proportion of citations predicted not relevant from the total

Of 1415 citations, 251 citations were screened (18 %) before Abstrackr made the predictions, leaving 1164 (82 %) citations unscreened. Of these, Abstrackr predicted that 374 citations were potentially relevant, and 63 were found to be relevant, giving a precision of 16.8 % (Fig. 1). The false negative rate was 10 % (Fig. 2). Of the 790 citations predicted not relevant, one citation was included in the review, giving a percentage missed of 0.13 % (Fig. 3). As 44 % of citations required screening and checking for relevance, a workload saving of 56 % was achieved (Fig. 4).

#### Sensitivity analysis of atypical haemolytic uremic syndrome dataset (including case reports)

The citations were re-screened using the same decisions to include or exclude citations—with the exception that case reports were included (even though irrelevant). This ‘homogeneous’ screening method increased the precision from 16.8 to 25.4 % (Fig. 1) and the false negative rate was 14.3 % (Fig. 2). The number of relevant citations missed, however, increased to two citations (0.21 %) (Fig. 3). The workload saving increased from 56 to 67 % (Fig. 3).

### Dietary fibre for colorectal cancer dataset

Of 517 citations, 120 citations (23 %) were screened before Abstrackr made predictions. Abstrackr predicted a further 190 were potentially relevant, and 47 were found to be relevant, giving a precision of 24.7 % (Fig. 1). The false negative rate was 14.5 % (Fig. 2). Of the remaining 207 citations predicted as not relevant by Abstrackr, none were included in the review—giving a 0 % missed (Fig. 3). Sixty percent of citations required screening and checking for relevance, providing a workload saving of 40 % (Fig. 4).

### Echocardiography for stroke dataset

Of 1735 citations, 122 (7 %) were screened before Abstrackr made predictions. Abstrackr predicted that a further 619 were potentially relevant, and 181 were found to be relevant giving a precision of 29.2 % (Fig. 1). The false negative rate was 4.7 % (Fig. 2). Of the remaining 994 citations predicted as not relevant by Abstrackr, 993 were correctly excluded; however, one citation that was included in the review was missed, giving a percentage missed of 0.10 % (Fig. 3). Forty-three percent of citations required screening and checking for relevance, providing a workload saving of 57 % (Fig. 4).

#### Sensitivity analysis of echocardiography for stroke (large dataset)

The citations were re-screened using a larger dataset of 15,920 citations to determine if precision and workload saving were affected. Abstrackr made predictions after 495 citations were screened and predicted that 2648 citations were potentially relevant. Of these, 181 were found to be relevant for full text inspection, giving a precision of 6.8 %. The false negative rate was 21.7 % (Fig. 2). Of the remaining 12,777 predicted as not relevant by Abstrackr, 12,775 were correctly predicted as not relevant. However, two citations that were included in the published review were missed, giving a percentage missed of 0.02 %. Twenty percent of citations required screening, providing a workload saving of 80 % (Fig. 4).

### Rituximab for Non-Hodgkin’s lymphoma

Of 1042 citations, 130 citations (12 %) were screened before Abstrackr made predictions. Abstrackr predicted 817 citations were potentially relevant, and 372 were found to be relevant giving a precision of 45.5 % (Fig. 1). The false negative rate was 2.4 % (Fig. 2). Of the remaining 95 citations predicted as not relevant by Abstrackr, none were included in the review, giving a percentage missed of 0 (Fig. 3). As 91 % of citations required screening and checking for relevance, there was only a 9 % workload saving (Fig. 4).

## Discussion

This study found that Abstrackr has the potential to reliably identify relevant citations and reduce workload from 9 to 80 %. In two datasets, all relevant citations were identified, and in the other two datasets, only one citation was missed. The false negative rate ranged from 2 to 21 %. Overall, precision was good although affected by the complexity of the review.

In the aHUS dataset, precision was only 16.8 %. This was due to the complexities of the study inclusion criteria which included case series as well as other higher quality study designs but not case reports that were excluded during screening. Because of the lexical similarity between case reports and case series, excluding case reports introduced greater variance into the machine learning algorithm with apparent conflicting decisions and consequently reduced precision. The sensitivity analysis demonstrated that by reducing ‘noise’, the precision could be increased. This problem of ‘noise’ with machine learning is common [21], and one strategy to increase precision during the data-training phase is to include close matching records [2], to ensure the active learning algorithm is not adversely affected, although this requires a degree of expertise to make decisions contrary to the PICOS (Participants, Interventions, Comparators, Outcomes and Study design) inclusion criteria. The ECHO sensitivity analysis had the worst precision (6.8 %) because of the 15,920 citations wherein there was only about 0.9 % that was relevant. Such imbalanced datasets are problematic for supervised machine learning models like Abstrackr, because the predictions are biased towards the majority non-relevant class at the expense of the minority-relevant class [22] and therefore produce many falsely weighted predictions, i.e. irrelevant citations. Nevertheless, this was off-set by the considerable workload saving.

The false negative rates ranged from 2 to 21.7 % and represent the percentage of citations that were relevant for further full text inspection but were predicted to be irrelevant by Abstrackr and were therefore ‘missed’. However, the actual percentage missed were in the range of 0 to 0.21 % and represent the true final proportion of citation missed by Abstrackr that were included in the review. Therefore, the classification model was almost completely reliable. The citation missed from the aHUS and ECHO datasets did not contain an abstract, only a title and therefore the probability of being predicted relevant was reduced. The aHUS sensitivity analysis missed two citations, and both contained no abstract. The ECHO sensitivity analysis missed two citations, one without an abstract, whilst the other did contain an abstract and it is unclear why this citation was not detected as relevant. However, these problems could be minimised by retaining citations without an abstract for manual inspection.

The complexity of the review PICOS criteria also affected the workload saving. The workload saving in the rituximab dataset was low (9 %) due to the rituximab intervention having multiple adjunctive chemotherapy treatments which overlapped with non-relevant studies. Therefore, the good precision and perfect recall accuracy with the rituximab data were off-set by the minimal workload savings suggesting that complex reviews may be less suited to semi-automated screening. Nevertheless, the average workload saving across the four datasets was 41 % and is similar to the findings reported by the developers of Abstrackr who achieved a 40 % saving in workload from two datasets [14].

Other data mining algorithms have achieved similar (40 %) workload savings [16] but recall (identifying relevant records) was lower (90 to 95 %), partly because testing was performed on datasets without a specifically associated research question. This makes comparisons with the results of this study difficult. Whilst another text mining algorithm [17] achieved workload savings ranging from 8.5 to 62 % with 15 test datasets, which are similar to our findings with Abstrackr (9 to 80 %), their results were based on a threshold of a minimum 95 % recall of relevant studies, which is too low for systematic reviews. The developers of Abstrackr reported a recall accuracy of 100 % for relevant studies from three genetics-related datasets and 99 % for a fourth dataset, whilst the average specificity across the four datasets was 87 % [14]. Their results were based on training the algorithm with balanced datasets, which have a similar number of relevant and irrelevant trials from the original systematic review, and using this trained algorithm to automatically find studies for the updates of the genetics-based systematic reviews. This approach is noteworthy since systematic reviews require update searches to be performed within 2 years of the first published version [23], therefore, implementing this strategy, by retaining the original classification model, would expedite the process of updating systematic reviews.

### Strengths and weaknesses of the research

Our findings may be limited by the four datasets used, and citations from other clinical specialities may yield different precision and workload saving and miss more relevant studies for inclusion, especially if the title and abstract descriptions are inadequate or the study designs are more complex. Our datasets were from recently published systematic reviews that included trials published mostly from 1995 onwards, and therefore, may contain better descriptions than older trials that were published before the CONSORT [24] reporting guidelines were introduced in 1996. Nevertheless, our results for identifying relevant trials are similar to the high recall results of Wallace (2010 and 2012) and indicate that similar accuracy could be achieved when using other datasets of medical citations. Previous text mining studies have mainly evaluated performance in terms of recall and specificity; however, our results also analysed the precision of the predictive model since this measures how precisely the algorithm selects studies for further full text inspection and mirrors the working steps of a systematic reviewer. Precision, however, is subjective and influenced by the reviewer’s expertise which can affect their screening judgements. The ECHO sensitivity analysis demonstrated that workload saving with semi-automated screening is more pronounced with large datasets, and therefore, greater savings could have resulted had we screened larger reviews. Nonetheless, the results provide a reasonable estimate of the algorithm’s typical performance during semi-automated screening.

This study and others that have evaluated semi-automated screening with support vector models [14, 15], semantic vector models [16], and complement naïve Bayes models [17] indicate that considerable workload savings can be achieved. The ability to identify all relevant citations with Abstrackr was very high but imperfect. Such accuracy, however, is acceptable as a stand-alone tool for scoping searches and non-systematic reviews where not every published study needs to be included. It is noteworthy, however, that human citation screening is imperfect with relevant studies wrongly excluded [25]. Given that Abstrackr’s inaccuracy is similar to a human screener, it could be utilised as the second screener. Abstrackr’s classification prediction model uses a somewhat arbitrary cutoff point at which the proportion of citations screened triggers the algorithm prediction. However, this suggests that an adjustable stopping heuristic could be used, so accuracy could be further improved albeit with the trade-off that more citations are screened during the training phase.

Future developments with semi-automated screening would benefit from retaining the original classification model developed during the original review, so future systematic review updates may be screened automatically without the re-input of a reviewer. Abstrackr is not currently a fully integrated tool, and only the unscreened citations (the predictions) are exportable with only the title bibliographic details made available, and further developments are needed to create a fully integrated tool that systematic reviewers and information specialists can use.

Text mining algorithms that would enable systematic reviewers to exploit the labelling of keywords towards biasing the predictive classification model to further enhance performance have been proposed [26]. This approach could be further aided by citation enrichment. For example, keywords of high relevance such as the PICOS details should improve the recall accuracy of semi-automated screening algorithms (and trial searching). Enriching citations is already being used for the EMBASE project [27] by coding citations with the type of study design through crowd sourcing. Further research and innovations in this underexplored area is needed to advance current methods to eventually enable semi-automated screening to fully replace manual screening. Current text mining research [28] is focused on advancing screening retroactively and is restrained by the limitations of the data available. A more successful approach may require collaboration with biomedical database providers to ensure that citations are adequately labelled prospectively and retrospectively using strategies such as record linkage techniques, crowd sourcing, or access to a central repository, whereby PICOS details can be inputted and linked to all bibliographic databases.

## Conclusions

Semi-automated screening with Abstrackr can potentially expedite the title and abstract screening phase of a systematic review. Although the accuracy is very high, relying solely on its predictions when used as a stand-alone tool is not yet possible. Nevertheless, efficiencies could still be attained by using Abstrackr as the second reviewer thereby saving time and resources.

## Abbreviations

aHUS: atypical hemolytic uremic syndrome
CENTRAL: The Cochrane Central Register of Controlled Trials
CINAHL: Cumulative Index to Nursing and Allied Health Literature
CONSORT: Consolidated Standards of Reporting Trials
ECHO: echocardiography
EMBASE: Excerpta Medica Database
MEDLINE: Medical Literature Analysis and Retrieval System Online
PICOS: Participants, Interventions, Comparators, Outcomes, Study design
